# Oxidative Stress in Postoperative Atrial Fibrillation: Does Malondialdehyde Hold Predictive Value?

**DOI:** 10.3390/medicina61050778

**Published:** 2025-04-22

**Authors:** Urska Intihar, Arta Krasniqi, Anze Djordjevic, Jan Zmazek, Harun Avdagic, Jus Ksela, Miha Antonic

**Affiliations:** 1 Department of Cardiac Surgery, University Medical Centre Maribor, Ljubljanska 5, 2000 Maribor, Slovenia; 2 Faculty of Natural Sciences and Mathematics, University of Maribor, 2000 Maribor, Slovenia; 3 Department of Cardiovascular Surgery, University Medical Centre Tuzla, 75000 Tuzla, Bosnia and Herzegovina; 4 Department of Cardiovascular Surgery, University Medical Centre Ljubljana, 1000 Ljubljana, Slovenia

**Keywords:** postoperative atrial fibrillation, oxidative stress, malondialdehyde, lipid peroxidation, cardiac surgery, biomarker, risk stratification, perioperative complications

## Abstract

*Background and Objectives*: Postoperative atrial fibrillation (POAF) is a common complication following cardiac surgery, associated with increased morbidity and prolonged hospital stays. Oxidative stress has been implicated in POAF pathogenesis, with malondialdehyde (MDA), a marker of lipid peroxidation, proposed as a potential biomarker. However, conflicting evidence exists regarding its predictive value. This study aimed to assess the association between serum MDA levels and POAF incidence in patients undergoing cardiac surgery. *Materials and Methods*: This prospective observational study included 99 consecutive patients undergoing elective on-pump cardiac surgery. Patients with preoperative atrial fibrillation, chronic kidney disease requiring dialysis, or emergency surgery were excluded. Blood samples for MDA measurement were collected at six perioperative time points: preoperatively, intraoperatively after aortic clamp release, and at 8, 24, 48, and 72 h postoperatively. Patients were monitored for new-onset POAF during the first three postoperative days. Statistical analyses included independent samples *t*-tests, Mann–Whitney U-tests, and Fisher’s exact tests, with significance set at *p* < 0.05. *Results*: POAF occurred in 33 (33%) patients. Patients who developed POAF were significantly older (*p* = 0.017) and had higher EuroSCORE II values (*p* = 0.019). No significant differences were observed in serum MDA concentrations between POAF and non-POAF patients at any measured time point. The incidence of POAF was higher in patients undergoing valvular surgery (*p* = 0.014). *Conclusions*: Serum MDA levels were not associated with POAF development, suggesting that lipid peroxidation alone may not play a central role in POAF pathogenesis. These findings challenge the predictive value of MDA for POAF risk stratification. Future research should explore alternative oxidative stress markers and their potential therapeutic implications in POAF prevention.

## 1. Introduction

Atrial fibrillation (AF) is the most common postoperative arrhythmia following cardiac surgery, with a reported incidence ranging from 20% to 50% in various patient populations [1]. Postoperative AF (POAF) is associated with increased morbidity, prolonged hospital stays, and a higher risk of stroke and mortality [2,3]. Despite extensive research advances in perioperative care, the precise pathophysiology of POAF remains incompletely elucidated [4]. Among various proposed contributors, oxidative stress—a key driver of cellular and tissue damage—has gained significant attention in promoting atrial structural and electrical remodelling.

Oxidative stress is characterized by an imbalance between reactive oxygen species (ROS) production and antioxidant defences, leading to cellular damage [5]. In the atrial myocardium, ROS can contribute to structural and electrical changes that predispose to fibrillation. In the perioperative setting, factors such as ischemia–reperfusion injury, systemic inflammation, and metabolic disturbances further exacerbate oxidative stress, increasing the likelihood of POAF development.

Malondialdehyde (MDA), a byproduct of lipid peroxidation, has been widely used as a biomarker of oxidative stress [6] and may provide insights into the pathophysiology and risk stratification of POAF [7]. In patients undergoing cardiac surgery, ischemia–reperfusion injury, exposure to cardiopulmonary bypass, and systemic inflammatory responses act as major contributors to oxidative stress and lipid peroxidation, leading to elevated MDA levels [8].

By synthesizing current evidence and identifying gaps in knowledge, this article seeks to provide a comprehensive understanding of the interplay between oxidative stress and POAF, focusing on the diagnostic and therapeutic potential of MDA.

## 2. Methods

This prospective observational study was conducted at the Department of Cardiac Surgery, University Medical Centre Maribor, Slovenia, over a six-month period from March to September 2019. This study was carried out in accordance with the ethical principles outlined in the Declaration of Helsinki, and written informed consent was obtained from all the participants prior to inclusion.

Eligible participants were adult patients scheduled for elective, on-pump open-heart surgery, including procedures such as coronary artery bypass grafting (CABG), aortic or mitral valve replacement/repair, or combined CABG and valve interventions. All surgeries were performed via median sternotomy using cardiopulmonary bypass (CPB) under a standardized anaesthesia protocol.

Patients were excluded if they underwent off-pump surgery, emergency or salvage procedures, required hypothermic circulatory arrest, had a critical preoperative status, or had preexisting atrial fibrillation. These criteria were applied to reduce confounding and maintain a uniform surgical and rhythmologic profile across the study population.

Blood samples for MDA measurement were collected at six perioperative time points: before skin incision, immediately after aortic cross-clamp release, and at 12, 24, 48, and 72 h postoperatively. These time points were selected based on prior studies that examined oxidative stress kinetics in cardiac surgery settings, aiming to capture both the acute oxidative response to ischemia–reperfusion and ongoing postoperative oxidative stress, which may be linked to the development of POAF [6,9,10].

In addition to serum collection, baseline demographic, clinical, and laboratory parameters were documented. Risk stratification was performed using the EuroSCORE II system. Intraoperative data, including CPB duration, aortic cross-clamp time, and urine output, were recorded. Continuous ECG monitoring was implemented from ICU admission through the postoperative hospital stay to identify new-onset POAF.

For MDA quantification, samples were centrifuged and derivatized with pentafluorophenylhydrazine (PFPH) at 50 °C for 10 min in sealed vials. The resulting derivatives were analysed using gas chromatography–mass spectrometry (GC–MS). Helium served as the carrier gas, and analytes were separated using a temperature-controlled gradient and detected via a mass spectrometer [6].

Statistical analyses were performed using IBM SPSS Statistics 25.0. Continuous variables were expressed as mean ± standard deviation (SD) or median [interquartile range], depending on distribution (assessed via the Shapiro–Wilk test). Categorical variables were presented as frequencies and percentages. Between-group comparisons were made using an independent samples *t*-test, the Mann–Whitney U test for continuous variables, and a chi-squared test for categorical variables. Statistical significance was defined as *p* < 0.05.

## 3. Results

A total of 99 patients were included in the trial. New-onset POAF occurred in 33/99 patients—an incidence rate of 33%. Table 1 shows the demographic and preoperative patient characteristics in our patient cohort. The two groups were comparable with regard to body mass index, preoperative haemoglobin, preoperative renal function measured via serum creatinine and estimated glomerular filtration rate (eGFR), and diabetes mellitus. Patients in the POAF group were older and had a higher EuroSCORE II. They also had a trend towards more significant peripheral atherosclerosis.

In the POAF group, the mean time from surgery to POAF onset was 4.35 ± 2.96 days, and most patients experienced an episode lasting less than 24 h (52%). All patients returned to normal sinus rhythm before discharge. The two groups were comparable with regard to the CPB time, aortic cross-clamp time, intraoperative urine output, and urgency. There were significantly more valvular procedures and a trend towards less coronary revascularization in the POAF group (Table 2). The average length of hospital stay for patients with POAF was 9.2 ± 2.6 days, compared to 8.3 ± 2.1 days in patients without POAF, which reflects a modest but not statistically significant increase (*p* = 0.062).

Table 3 compares serum MDA concentrations in both groups on the abovementioned time points. There were no significant differences between the two groups.

## 4. Discussion

The main finding of this study is that serum malondialdehyde levels, a well-established biomarker of oxidative stress, do not significantly differ between patients who develop postoperative atrial fibrillation and those who do not. These results suggest that MDA may not be a reliable predictor of POAF occurrence in patients undergoing cardiac surgery. Given the known role of oxidative stress in atrial fibrillation pathogenesis, this finding is somewhat unexpected and warrants further exploration.

The pathogenesis of POAF after cardiac surgery is complex and multifactorial. Probable etiologic factors include intraoperative manipulation of the heart, surgical trauma to the atria, and local ischemia. Systemic factors such as sympathetic activation, systemic inflammation, and electrolyte disturbance have also been implicated in the genesis of POAF. Recent studies have suggested a potential link between elevated MDA levels and the incidence of POAF, positioning MDA as a promising biomarker for risk stratification and early intervention [11,12]. Despite promising preclinical data, the efficacy of antioxidant therapy in reducing perioperative AF remains controversial, warranting further investigation into targeted oxidative stress modulation strategies [6].

POAF remains a frequent complication after cardiac surgery, occurring in 33% of our study population. Consistent with previous literature [2,4], we observed that patients who developed POAF were significantly older and had higher EuroSCORE II values, reflecting a greater overall surgical risk. While oxidative stress has been implicated as a key factor in atrial electrical and structural remodelling [13,14,15], our study did not find a direct association between elevated MDA levels and POAF incidence. This contrasts with several prior studies suggesting that increased preoperative or postoperative MDA concentrations correlate with a higher risk of POAF [9,16]. One possible explanation for this discrepancy is the timing of the MDA measurement. While we collected MDA levels at multiple perioperative time points, oxidative stress is a dynamic process influenced by various intraoperative and postoperative factors, including systemic inflammation, ischemia–reperfusion injury, and patient-specific antioxidant defences. It is possible that transient spikes in oxidative stress markers, rather than absolute MDA values at predefined time points, better predict POAF onset.

However, some data support our results. In a recent study by McDonald and colleagues [10], the effect of low preoperative selenium levels on the incidence of postoperative atrial fibrillation was investigated in 50 cardiac surgical patients. The MDA levels were measured preoperatively, after the aortic cross-clamp removal, three hours after the surgery, and on postoperative days 1 and 5. The MDA concentrations in patients who developed postoperative atrial fibrillation were then compared to patients without postoperative atrial fibrillation. Patients with postoperative atrial fibrillation had similar plasma levels of MDA preoperatively and postoperatively until postoperative day 5, when their MDA concentrations were significantly higher than in patients without atrial fibrillation. Caldonazo et al. [17] conducted a meta-analysis and found that while some included studies showed higher MDA levels in POAF patients, several did not. They have concluded that MDA is not a consistent independent predictor of POAF across studies and recommended looking at multiple oxidative stress markers together rather than relying on MDA alone.

Another notable observation was the higher prevalence of valvular surgery in the POAF group, consistent with existing literature [18,19]. While we did not stratify results by specific valve types or procedure characteristics due to sample size constraints, prior studies have shown that mitral valve surgeries, in particular, carry a higher risk of POAF due to extensive atrial manipulation and volume overload. Future research with larger cohorts should explore the influence of valve type and intervention method on POAF risk.

Despite our findings, it remains plausible that oxidative stress contributes to POAF through mechanisms beyond lipid peroxidation. Reactive oxygen species (ROS) and other oxidative damage pathways, such as protein oxidation or mitochondrial dysfunction, may play more central roles. Additionally, individual variability in antioxidant enzyme activity and systemic inflammatory responses could modulate oxidative stress effects on atrial arrhythmogenesis, potentially explaining why some patients with similar MDA levels developed POAF while others did not.

It is well-established that patients with diabetes mellitus and peripheral artery disease often exhibit elevated MDA levels due to chronic oxidative stress [20,21]. We conducted subgroup analyses excluding patients with either condition to rule out confounding effects. The findings remained consistent, with no significant differences in MDA levels between POAF and non-POAF groups, suggesting that the lack of association was not attributable to these comorbidities. This reinforces the conclusion that MDA may not independently predict POAF in the context of cardiac surgery.

Although our findings suggest that MDA alone may not serve as a reliable standalone biomarker for predicting POAF, this study contributes to a growing body of evidence on the role of oxidative stress in cardiac surgery patients. Future research could explore how MDA, in combination with other oxidative or inflammatory markers, may be incorporated into multi-parameter risk stratification models. Such models could aid in identifying high-risk individuals preoperatively and tailoring perioperative management strategies accordingly. For instance, patients identified as having elevated oxidative stress profiles could potentially benefit from antioxidant therapy, closer postoperative rhythm monitoring, or earlier pharmacologic prophylaxis to mitigate the risk of POAF. These strategies could enhance patient outcomes by reducing POAF incidence and its associated complications.

Several limitations must be acknowledged. First, the relatively small sample size (99 patients) may have limited the statistical power to detect subtle differences in MDA levels between groups. A larger cohort might reveal more nuanced oxidative stress patterns associated with POAF. Second, while MDA is a widely used biomarker of lipid peroxidation, it does not capture the full spectrum of oxidative or inflammatory responses associated with POAF. Future studies incorporating complementary biomarkers—such as CRP, NT-proBNP, and troponin [22,23,24,25]—may provide a more comprehensive understanding of the molecular mechanisms driving POAF and enhance the predictive value of biomarker-based risk stratification. Third, we did not assess genetic or pharmacological factors that could influence oxidative stress responses, such as polymorphisms in antioxidant enzyme genes or preoperative use of statins, beta-blockers, or antioxidants [26,27], which may have confounded the results. Lastly, while our study design ensured serial MDA measurements at key perioperative time points, continuous monitoring of oxidative stress markers could provide more precise insights into their role in POAF pathogenesis.

In conclusion, our study suggests that MDA alone may not serve as a reliable biomarker for POAF prediction in cardiac surgery patients. Future research should explore alternative oxidative stress markers and investigate whether antioxidant therapies targeting broader oxidative pathways could mitigate POAF risk.

## Figures and Tables

**Table 1:** Preoperative patient characteristics.

	POAF (*n* = 33)	No POAF (*n* = 66)	*p*-Value
Age (years)	72 [65–80]	65.5 [60–73]	0.017
Body mass index (kg/m^2^)	30.0 [26.8–34.1]	27.0 [23.8–31.4]	0.157
Haemoglobin (g/L)	133.91 ± 14.48	136.18 ± 17.07	0.570
EuroSCORE II (%)	2.55 [1.25–4.37]	1.53 [0.98–2.63]	0.019
Creatinine (µmol/L)	81.0 [72.0–90.0]	82.5 [70.0–95.8]	0.620
eGFR (mL/min/1.73 m^2^)	73.00 ± 16.75	78.79 ± 25.73	0.202
Diabetes mellitus	4 (12%)	16 (24%)	0.157
Peripheral artery disease	13 (39%)	14 (21%)	0.056

EuroSCORE: European System for Cardiac Operative Risk Evaluation; eGFR: estimated glomerular filtration rate.

**Table 2:** Intraoperative data.

	POAF (*n* = 33)	No POAF (*n* = 66)	*p*-Value
CPB time (min)	118.97 ± 43.18	103.80 ± 30.34	0.129
Aortic cross-clamp time (min)	92.91 ± 33.21	82.37 ± 23.12	0.164
CPB urine output (ml)	339.03 ± 208.35	331.72 ± 184.73	0.808
CABG (%)	17 (52)	45 (68)	0.059
Valvular surgery (%)	22 (67)	26 (39)	0.014
Aortic surgery (%)	2 (6)	4 (6)	0.970
Urgent surgery (%)	13 (39)	22 (33)	0.552

CPB: cardiopulmonary bypass; CABG: coronary artery bypass grafting.

**Table 3:** Malondialdehyde dynamics.

	POAF (*n* = 33)	No POAF (*n* = 66)	*p*-Value
MDA before surgery (ng/mL)	23.29 ± 17.09	19.57 ± 16.50	0.300
MDA immediately after surgery (ng/mL)	40.80 ± 42.82	36.11 ± 32.75	0.997
MDA 8 h after surgery (ng/mL)	37.81 ± 34.01	38.87 ± 35.26	0.903
MDA POD 1 (ng/mL)	45.56 ± 44.43	38.02 ± 30.13	0.883
MDA POD 2 (ng/mL)	37.91 ± 33.46	33.86 ± 29.17	0.718
MDA POD 3 (ng/mL)	29.46 ± 32.74	23.39 ± 25.13	0.638

MDA: malondialdehyde.

## Data Availability

The original contributions presented in this study are included in the article. Further inquiries can be directed to the corresponding author.
